# Supplementary material for: Resolving the full spectrum of human genome variation using Linked-Reads
Source: Genome Res. 2019 Apr;29(4):635–45. doi: 10.1101/gr.234443.118 (PMC6442396; doi:10.1101/gr.234443.118)
Supplement: Supplemental Material [file supp_gr.234443.118_Supplemental_longranger-2.2.zip › longranger-2.2.tar.bz2!/lr2.2-public/lib/stan/cmdstan/stan/doc/api/html/rayleigh_8hpp_source.html]

Stan: src/stan/prob/distributions/univariate/continuous/rayleigh.hpp Source File


|  |  |
| --- | --- |
|  | Stan  2.5.0  probability, sampling & optimization |


- Main Page
- Related Pages
- Namespaces
- Classes
- Files

- File List
- File Members

All Classes Namespaces Files Functions Variables Typedefs Enumerations Enumerator Friends Macros Pages

- src
- stan
- prob
- distributions
- univariate
- continuous

rayleigh.hpp

Go to the documentation of this file.

1 #ifndef STAN\_\_PROB\_\_DISTRIBUTIONS\_\_UNIVARIATE\_\_CONTINUOUS\_\_RAYLEIGH\_HPP

2 #define STAN\_\_PROB\_\_DISTRIBUTIONS\_\_UNIVARIATE\_\_CONTINUOUS\_\_RAYLEIGH\_HPP

3

4 #include <boost/random/uniform\_real\_distribution.hpp>

5 #include <boost/random/variate\_generator.hpp>

6

7 #include <stan/agrad/partials\_vari.hpp>

8 #include <stan/math.hpp>

9 #include <stan/math/error\_handling.hpp>

10 #include <stan/meta/traits.hpp>

11 #include <stan/prob/constants.hpp>

12 #include <stan/prob/traits.hpp>

13

14 namespace stan {

15

16  namespace prob {

17

18  template <bool propto,

19  typename T\_y, typename T\_scale>

20  typename return\_type<T\_y,T\_scale>::type

21  rayleigh\_log(const T\_y& y, const T\_scale& sigma) {

22  static const char\* function = "stan::prob::rayleigh\_log(%1%)";

23

24  using std::log;

25  using stan::is\_constant\_struct;

26  using stan::math::check\_positive;

27  using stan::math::check\_not\_nan;

28  using stan::math::check\_consistent\_sizes;

29  using stan::math::value\_of;

30  using stan::prob::include\_summand;

31

32  // check if any vectors are zero length

33  if (!(stan::length(y) && stan::length(sigma)))

34  return 0.0;

35

36  // set up return value accumulator

37  double logp(0.0);

38

39  // validate args (here done over var, which should be OK)

40  check\_not\_nan(function, y, "Random variable", &logp);

41  check\_positive(function, sigma, "Scale parameter", &logp);

42  check\_positive(function, y, "Random variable", &logp);

43  check\_consistent\_sizes(function,

44  y,sigma,

45  "Random variable","Scale parameter",

46  &logp);

47

48  // check if no variables are involved and prop-to

49  if (!include\_summand<propto,T\_y,T\_scale>::value)

50  return 0.0;

51

52  // set up template expressions wrapping scalars into vector views

53  agrad::OperandsAndPartials<T\_y, T\_scale> operands\_and\_partials(y, sigma);

54

55  VectorView<const T\_y> y\_vec(y);

56  VectorView<const T\_scale> sigma\_vec(sigma);

57  size\_t N = max\_size(y, sigma);

58

59  DoubleVectorView<true,is\_vector<T\_scale>::value> inv\_sigma(length(sigma));

60  DoubleVectorView<include\_summand<propto,T\_scale>::value,

61  is\_vector<T\_scale>::value> log\_sigma(length(sigma));

62  for (size\_t i = 0; i < length(sigma); i++) {

63  inv\_sigma[i] = 1.0 / value\_of(sigma\_vec[i]);

64  if (include\_summand<propto,T\_scale>::value)

65  log\_sigma[i] = log(value\_of(sigma\_vec[i]));

66  }

67

68  for (size\_t n = 0; n < N; n++) {

69  // pull out values of arguments

70  const double y\_dbl = value\_of(y\_vec[n]);

71

72  // reusable subexpression values

73  const double y\_over\_sigma = y\_dbl \* inv\_sigma[n];

74

75  static double NEGATIVE\_HALF = -0.5;

76

77  // log probability

78  if (include\_summand<propto,T\_scale>::value)

79  logp -= 2.0 \* log\_sigma[n];

80  if (include\_summand<propto,T\_y>::value)

81  logp += log(y\_dbl);

82  // if (include\_summand<propto,T\_y,T\_scale>::value)

83  logp += NEGATIVE\_HALF \* y\_over\_sigma \* y\_over\_sigma;

84

85  // gradients

86  double scaled\_diff = inv\_sigma[n] \* y\_over\_sigma;

87  if (!is\_constant\_struct<T\_y>::value)

88  operands\_and\_partials.d\_x1[n] += 1.0 / y\_dbl - scaled\_diff;

89  if (!is\_constant\_struct<T\_scale>::value)

90  operands\_and\_partials.d\_x2[n]

91  += y\_over\_sigma \* scaled\_diff - 2.0 \* inv\_sigma[n];

92  }

93  return operands\_and\_partials.to\_var(logp);

94  }

95

96  template <typename T\_y, typename T\_scale>

97  inline

98  typename return\_type<T\_y,T\_scale>::type

99  rayleigh\_log(const T\_y& y, const T\_scale& sigma) {

100  return rayleigh\_log<false>(y,sigma);

101  }

102

103  template <typename T\_y, typename T\_scale>

104  typename return\_type<T\_y,T\_scale>::type

105  rayleigh\_cdf(const T\_y& y, const T\_scale& sigma) {

106  static const char\* function = "stan::prob::rayleigh\_cdf(%1%)";

107

108  using stan::math::check\_nonnegative;

109  using stan::math::check\_positive;

110  using stan::math::check\_not\_nan;

111  using stan::math::check\_consistent\_sizes;

112  using stan::prob::include\_summand;

113  using stan::is\_constant\_struct;

114  using stan::math::square;

115  using stan::math::value\_of;

116

117  double cdf(1.0);

118

119  // check if any vectors are zero length

120  if (!(stan::length(y) && stan::length(sigma)))

121  return cdf;

122

123  check\_not\_nan(function, y, "Random variable", &cdf);

124  check\_nonnegative(function, y, "Random variable", &cdf);

125  check\_not\_nan(function, sigma, "Scale parameter", &cdf);

126  check\_positive(function, sigma, "Scale parameter", &cdf);

127  check\_consistent\_sizes(function,

128  y,sigma,

129  "Random variable","Scale parameter",

130  &cdf);

131

132

133  // set up template expressions wrapping scalars into vector views

134  agrad::OperandsAndPartials<T\_y, T\_scale> operands\_and\_partials(y, sigma);

135

136  VectorView<const T\_y> y\_vec(y);

137  VectorView<const T\_scale> sigma\_vec(sigma);

138  size\_t N = max\_size(y, sigma);

139

140  DoubleVectorView<true,is\_vector<T\_scale>::value> inv\_sigma(length(sigma));

141  for (size\_t i = 0; i < length(sigma); i++) {

142  inv\_sigma[i] = 1.0 / value\_of(sigma\_vec[i]);

143  }

144

145  for (size\_t n = 0; n < N; n++) {

146  const double y\_dbl = value\_of(y\_vec[n]);

147  const double y\_sqr = y\_dbl \* y\_dbl;

148  const double inv\_sigma\_sqr = inv\_sigma[n] \* inv\_sigma[n];

149  const double exp\_val = exp(-0.5 \* y\_sqr \* inv\_sigma\_sqr);

150

151  if (include\_summand<false,T\_y,T\_scale>::value)

152  cdf \*= (1.0 - exp\_val);

153  }

154

155  //gradients

156  for (size\_t n = 0; n < N; n++) {

157  const double y\_dbl = value\_of(y\_vec[n]);

158  const double y\_sqr = square(y\_dbl);

159  const double inv\_sigma\_sqr = square(inv\_sigma[n]);

160  const double exp\_val = exp(-0.5 \* y\_sqr \* inv\_sigma\_sqr);

161  const double exp\_div\_1m\_exp = exp\_val / (1.0 - exp\_val);

162

163  if (!is\_constant\_struct<T\_y>::value)

164  operands\_and\_partials.d\_x1[n] += y\_dbl \* inv\_sigma\_sqr

165  \* exp\_div\_1m\_exp \* cdf;

166  if (!is\_constant\_struct<T\_scale>::value)

167  operands\_and\_partials.d\_x2[n] -= y\_sqr \* inv\_sigma\_sqr

168  \* inv\_sigma[n] \* exp\_div\_1m\_exp \* cdf;

169  }

170

171  return operands\_and\_partials.to\_var(cdf);

172  }

173

174  template <typename T\_y, typename T\_scale>

175  typename return\_type<T\_y,T\_scale>::type

176  rayleigh\_cdf\_log(const T\_y& y, const T\_scale& sigma) {

177  static const char\* function = "stan::prob::rayleigh\_cdf\_log(%1%)";

178

179  using stan::math::check\_nonnegative;

180  using stan::math::check\_positive;

181  using stan::math::check\_not\_nan;

182  using stan::math::check\_consistent\_sizes;

183  using stan::prob::include\_summand;

184  using stan::is\_constant\_struct;

185  using stan::math::square;

186  using stan::math::value\_of;

187

188  double cdf\_log(0.0);

189

190  // check if any vectors are zero length

191  if (!(stan::length(y) && stan::length(sigma)))

192  return cdf\_log;

193

194  check\_not\_nan(function, y, "Random variable", &cdf\_log);

195  check\_nonnegative(function, y, "Random variable", &cdf\_log);

196  check\_not\_nan(function, sigma, "Scale parameter", &cdf\_log);

197  check\_positive(function, sigma, "Scale parameter", &cdf\_log);

198  check\_consistent\_sizes(function,

199  y,sigma,

200  "Random variable","Scale parameter",

201  &cdf\_log);

202

203

204  // set up template expressions wrapping scalars into vector views

205  agrad::OperandsAndPartials<T\_y, T\_scale> operands\_and\_partials(y, sigma);

206

207  VectorView<const T\_y> y\_vec(y);

208  VectorView<const T\_scale> sigma\_vec(sigma);

209  size\_t N = max\_size(y, sigma);

210

211  DoubleVectorView<true,is\_vector<T\_scale>::value> inv\_sigma(length(sigma));

212  for (size\_t i = 0; i < length(sigma); i++) {

213  inv\_sigma[i] = 1.0 / value\_of(sigma\_vec[i]);

214  }

215

216  for (size\_t n = 0; n < N; n++) {

217  const double y\_dbl = value\_of(y\_vec[n]);

218  const double y\_sqr = y\_dbl \* y\_dbl;

219  const double inv\_sigma\_sqr = inv\_sigma[n] \* inv\_sigma[n];

220  const double exp\_val = exp(-0.5 \* y\_sqr \* inv\_sigma\_sqr);

221  const double exp\_div\_1m\_exp = exp\_val / (1.0 - exp\_val);

222

223  if (include\_summand<false,T\_y,T\_scale>::value)

224  cdf\_log += log(1.0 - exp\_val);

225

226  if (!is\_constant\_struct<T\_y>::value)

227  operands\_and\_partials.d\_x1[n] += y\_dbl \* inv\_sigma\_sqr

228  \* exp\_div\_1m\_exp;

229  if (!is\_constant\_struct<T\_scale>::value)

230  operands\_and\_partials.d\_x2[n] -= y\_sqr \* inv\_sigma\_sqr

231  \* inv\_sigma[n] \* exp\_div\_1m\_exp;

232  }

233

234  return operands\_and\_partials.to\_var(cdf\_log);

235  }

236

237  template <typename T\_y, typename T\_scale>

238  typename return\_type<T\_y,T\_scale>::type

239  rayleigh\_ccdf\_log(const T\_y& y, const T\_scale& sigma) {

240  static const char\* function = "stan::prob::rayleigh\_ccdf\_log(%1%)";

241

242  using stan::math::check\_nonnegative;

243  using stan::math::check\_positive;

244  using stan::math::check\_not\_nan;

245  using stan::math::check\_consistent\_sizes;

246  using stan::prob::include\_summand;

247  using stan::is\_constant\_struct;

248  using stan::math::square;

249  using stan::math::value\_of;

250

251  double ccdf\_log(0.0);

252

253  // check if any vectors are zero length

254  if (!(stan::length(y) && stan::length(sigma)))

255  return ccdf\_log;

256

257  check\_not\_nan(function, y, "Random variable", &ccdf\_log);

258  check\_nonnegative(function, y, "Random variable", &ccdf\_log);

259  check\_not\_nan(function, sigma, "Scale parameter", &ccdf\_log);

260  check\_positive(function, sigma, "Scale parameter", &ccdf\_log);

261  check\_consistent\_sizes(function,

262  y,sigma,

263  "Random variable","Scale parameter",

264  &ccdf\_log);

265

266

267  // set up template expressions wrapping scalars into vector views

268  agrad::OperandsAndPartials<T\_y, T\_scale> operands\_and\_partials(y, sigma);

269

270  VectorView<const T\_y> y\_vec(y);

271  VectorView<const T\_scale> sigma\_vec(sigma);

272  size\_t N = max\_size(y, sigma);

273

274  DoubleVectorView<true,is\_vector<T\_scale>::value> inv\_sigma(length(sigma));

275  for (size\_t i = 0; i < length(sigma); i++) {

276  inv\_sigma[i] = 1.0 / value\_of(sigma\_vec[i]);

277  }

278

279  for (size\_t n = 0; n < N; n++) {

280  const double y\_dbl = value\_of(y\_vec[n]);

281  const double y\_sqr = y\_dbl \* y\_dbl;

282  const double inv\_sigma\_sqr = inv\_sigma[n] \* inv\_sigma[n];

283

284  if (include\_summand<false,T\_y,T\_scale>::value)

285  ccdf\_log += -0.5 \* y\_sqr \* inv\_sigma\_sqr;

286

287  if (!is\_constant\_struct<T\_y>::value)

288  operands\_and\_partials.d\_x1[n] -= y\_dbl \* inv\_sigma\_sqr;

289  if (!is\_constant\_struct<T\_scale>::value)

290  operands\_and\_partials.d\_x2[n] += y\_sqr \* inv\_sigma\_sqr

291  \* inv\_sigma[n];

292  }

293

294  return operands\_and\_partials.to\_var(ccdf\_log);

295  }

296

297  template <class RNG>

298  inline double

299  rayleigh\_rng(const double sigma,

300  RNG& rng) {

301  using boost::variate\_generator;

302  using boost::random::uniform\_real\_distribution;

303

304  static const char\* function = "stan::prob::rayleigh\_rng(%1%)";

305

306  using stan::math::check\_positive;

307

308  check\_positive(function, sigma, "Scale parameter", (double\*)0);

309

310  variate\_generator<RNG&, uniform\_real\_distribution<> >

311  uniform\_rng(rng, uniform\_real\_distribution<>(0.0, 1.0));

312  return sigma \* std::sqrt(-2.0 \* std::log(uniform\_rng()));

313  }

314  }

315 }

316 #endif

stan::math::square

T square(const T x)

Return the square of the specified argument.

**Definition:** square.hpp:22

traits.hpp

stan::prob::rayleigh\_cdf

return\_type< T\_y, T\_scale >::type rayleigh\_cdf(const T\_y &y, const T\_scale &sigma)

**Definition:** rayleigh.hpp:105

stan::agrad::OperandsAndPartials::to\_var

T\_return\_type to\_var(double logp)

**Definition:** partials\_vari.hpp:138

error\_handling.hpp

stan::length

size\_t length(const T &)

**Definition:** traits.hpp:159

stan::agrad::square

fvar< T > square(const fvar< T > &x)

**Definition:** square.hpp:15

stan::DoubleVectorView

DoubleVectorView allocates double values to be used as intermediate values.

**Definition:** traits.hpp:358

partials\_vari.hpp

stan::agrad::value\_of

T value\_of(const fvar< T > &v)

Return the value of the specified variable.

**Definition:** value\_of.hpp:16

constants.hpp

stan::is\_vector

**Definition:** traits.hpp:83

stan::agrad::OperandsAndPartials

A variable implementation that stores operands and derivatives with respect to the variable...

**Definition:** partials\_vari.hpp:76

traits.hpp

stan::return\_type::type

boost::math::tools::promote\_args< typename scalar\_type< T1 >::type, typename scalar\_type< T2 >::type, typename scalar\_type< T3 >::type, typename scalar\_type< T4 >::type, typename scalar\_type< T5 >::type, typename scalar\_type< T6 >::type >::type type

**Definition:** traits.hpp:406

stan::prob::rayleigh\_ccdf\_log

return\_type< T\_y, T\_scale >::type rayleigh\_ccdf\_log(const T\_y &y, const T\_scale &sigma)

**Definition:** rayleigh.hpp:239

stan::is\_constant\_struct

Metaprogram to determine if a type has a base scalar type that can be assigned to type double...

**Definition:** traits.hpp:57

stan::math::value\_of

double value\_of(const T x)

Return the value of the specified scalar argument converted to a double value.

**Definition:** value\_of.hpp:24

stan::prob::include\_summand

Template metaprogram to calculate whether a summand needs to be included in a proportional (log) prob...

**Definition:** traits.hpp:35

math.hpp

stan::agrad::sqrt

fvar< T > sqrt(const fvar< T > &x)

**Definition:** sqrt.hpp:15

stan::agrad::OperandsAndPartials::d\_x2

VectorView< double \*, is\_vector< T2 >::value, is\_constant\_struct< T2 >::value > d\_x2

**Definition:** partials\_vari.hpp:83

stan::prob::uniform\_rng

double uniform\_rng(const double alpha, const double beta, RNG &rng)

**Definition:** uniform.hpp:339

stan::math::check\_nonnegative

bool check\_nonnegative(const char \*function, const T\_y &y, const char \*name, T\_result \*result)

**Definition:** check\_nonnegative.hpp:52

stan::math::check\_consistent\_sizes

bool check\_consistent\_sizes(const char \*function, const T1 &x1, const T2 &x2, const char \*name1, const char \*name2, T\_result \*result)

**Definition:** check\_consistent\_sizes.hpp:13

stan::max\_size

size\_t max\_size(const T1 &x1, const T2 &x2)

**Definition:** traits.hpp:191

stan::math::check\_positive

bool check\_positive(const char \*function, const T\_y &y, const char \*name, T\_result \*result)

**Definition:** check\_positive.hpp:54

stan::prob::rayleigh\_cdf\_log

return\_type< T\_y, T\_scale >::type rayleigh\_cdf\_log(const T\_y &y, const T\_scale &sigma)

**Definition:** rayleigh.hpp:176

stan::prob::rayleigh\_log

return\_type< T\_y, T\_scale >::type rayleigh\_log(const T\_y &y, const T\_scale &sigma)

**Definition:** rayleigh.hpp:21

stan::math::check\_not\_nan

bool check\_not\_nan(const char \*function, const T\_y &y, const char \*name, T\_result \*result)

Checks if the variable y is nan.

**Definition:** check\_not\_nan.hpp:56

stan::agrad::OperandsAndPartials::d\_x1

VectorView< double \*, is\_vector< T1 >::value, is\_constant\_struct< T1 >::value > d\_x1

**Definition:** partials\_vari.hpp:82

stan::prob::rayleigh\_rng

double rayleigh\_rng(const double sigma, RNG &rng)

**Definition:** rayleigh.hpp:299

stan::agrad::log

fvar< T > log(const fvar< T > &x)

**Definition:** log.hpp:15

stan::VectorView

VectorView is a template metaprogram that takes its argument and allows it to be used like a vector...

**Definition:** traits.hpp:275

stan::agrad::exp

fvar< T > exp(const fvar< T > &x)

**Definition:** exp.hpp:16


---

[ Stan Home Page ]

*© 2011–2014,
Stan Development Team.*
